# Autophagy-Modulated Human Bone Marrow-Derived Mesenchymal Stem Cells Accelerate Liver Restoration in Mouse Models of Acute Liver Failure

**DOI:** 10.7508/ibj.2016.03.002

**Published:** 2016-07

**Authors:** Fatemeh Amiri, Sedigheh Molaei, Marzie Bahadori, Fatemeh Nasiri, Mohammad Reza Deyhim, Mohammad Ali Jalili, Mohammad Reza Nourani, Mehryar Habibi Roudkenar

**Affiliations:** 1 Blood Transfusion Research Center, High Institute for Research and Education in Transfusion Medicine, Tehran, Iran;; 2 School of Medicine, Qum University of Medical Sciences, Qum, Iran;; 3 Research Center of Molecular Biology, Baqiyatallah Medical Sciences University, Tehran, Iran

**Keywords:** Acute liver failure, Mesenchymal stem cells, Autophagy

## Abstract

**Background:**

Mesenchymal stem cells (MSCs) have been recently received increasing attention for cell-based therapy, especially in regenerative medicine. However, the low survival rate of these cells restricts their therapeutic applications. It is hypothesized that autophagy might play an important role in cellular homeostasis and survival. This study aims to investigate the regenerative potentials of autophagy-modulated MSCs for the treatment of acute liver failure (ALF) in mice.

**Methods:**

ALF was induced in mice by intraperitoneal injection of 1.5 ml/kg carbon tetrachloride. Mice were intravenously infused with MSCs, which were suppressed in their autophagy pathway. Blood and liver samples were collected at different intervals (24, 48 and 72 h) after the transplantation of MSCs. Both the liver enzymes and tissue necrosis levels were evaluated using biochemical and histopathological assessments. The survival rate of the transplanted mice was also recorded during one week.

**Results:**

Biochemical and pathological results indicated that 1.5 ml/kg carbon tetrachloride induces ALF in mice. A significant reduction of liver enzymes and necrosis score were observed in autophagy-modulated MSC-transplanted mice compared to sham (with no cell therapy) after 24 h. After 72 h, liver enzymes reached their normal levels in mice transplanted with autophagy-suppressed MSCs. Interestingly, normal histology without necrosis was also observed.

**Conclusion:**

Autophagy suppression in MSCs ameliorates their liver regeneration potentials due to paracrine effects and might be suggested as a new strategy for the improvement of cell therapy in ALF.

## INTRODUCTION

Mesenchymal stem cells (MSCs) are multipotent stem cells isolated from different tissues and easily expanded *in vitro*^[^^1^^]^. These cells have several main features, including self-renewal, adherence to plastic, differentiation into at least three types of mesodermal layers (osteocytes, adipocytes and chondrocytes), expression of main MSC markers (CD105, CD90, CD29 and CD73) and lack of hematopoietic/endothelial markers expression^[^^2^^]^. MSCs secrete several types of cytokines, chemokines and growth factors involved in cell proliferation and differentiation^[^^3^^]^. Moreover, MSCs are known as immunomodulatory cells because of the low expression of major histocompatibility complex antigens on their cellular surface and production of anti-inflammatory molecules^[^^4^^]^. Paracrine ability of MSCs along with MSCs-specific receptors and ligands expression profiles contributes to MSC migration toward damaged tissues^[^^5^^,^^6^^]^, where they start homing and recover damaged tissues using their immune-modulatory and trophic functions^[^^7^^]^. Regarding these promising characteristics, MSCs have been employed in a wide variety of damages and disease experimental models in order to discover their definite curative potentials^[^^8^^]^. 

In the last decades, MSCs have also been suggested for treatment of liver diseases, such as acute liver failure (ALF), cirrhosis and fibrosis^[^^9^^-^^11^^]^. ALF, a liver tissue injury with extensive necrosis, is induced by drugs, infections, toxins and chemicals and associated with the high rates of morbidity and mortality^[^^12^^]^. Liver transplantation is the only curative treatment for ALF^[^^13^^]^. Unfortunately, the low number of donated liver is the most important limitation of liver transplantation^[^^14^^]^. Hence, treatment strategies such as MSC-based therapeutic approaches have been proposed as an alternative to whole organ transplantation^[^^8^^,^^10^^-^^11^^,^^15^^]^. However, MSC-based cell therapy has been hindered mostly because of some limitations such as restricted lifespan and low cellular survival rate of MSCs^[^^16^^]^. Therefore, addressing this challenge is under the focus of investigations. Preconditioning under hypoxic conditions such as H_2_O_2_ (oxidative stress) and pretreatment of MSCs with special cytokines have been some strategies frequently employed to improve MSCs survival and potentialities in recent years^[^^17^^]^. 

Autophagy, a catabolic process of garbage disposal system, plays an important role in cell homeostasis and survival regarding the cell type and context^[^^18^^]^. Autophagy might act in either cellular death or survival through knocking down of different autophagy genes^[^^19^^,^^20^^]^. Therefore, the precise role of autophagy is still controversial. Here, we focused on *ATG7*, which is a key autophagy gene. Modulation of *ATG7* expression seems to show some correlations with MSCs survival and potentials; however, few practical studies have been performed in this regard. 

Interestingly, in our recent *in vitro *study, we have shown that the suppression of autophagy in MSCs enhances their survival rate^[^^21^^]^. To further expand our previous findings, herein, we examined whether the autophagy-modulated MSCs would facilitate and accelerate liver recovery after the induction of mice ALF models. Moreover, MSCs can be isolated from many different sources but in this study we used human bone marrow-derived MSCs due to their simple availability and their potentiality of being employed in clinical trials. 

## MATERIALS AND METHODS

***In vitro*** **studies**

***The preparation of mesenchymal stem cells*** ***and in vitro treatments***

To prepare different MSC groups (autophagy-modulated MSCs and normal MSCs), MSCs were isolated from human bone marrow aspirate based on their adherent character^[^^22^^]^. The bone marrow aspirates were collected from healthy male volunteers with an informed consent. Passages 3-5 of MSCs were expanded in Dulbecco's modified eagle's medium low-glucose medium (Invitrogen, USA) supplemented with 10% fetal bovine serum and 1% penicillin and streptomycin antibiotics (all from Invitrogen, USA). 

*ATG7*, a key autophagy gene, was knocked down using specific SureSilencing short hairpin RNA (*ATG7*-shRNA) suppressing vector in order to suppress autophagy in MSCs^[^^21^^]^. Briefly, MSCs were transfected with the suppressing vectors using a FuGENE HD transfection reagent (Roche, Germany), and the reduction of *ATG7* expression was evaluated by RT-PCR and real-time PCR. The inhibitory effects of *ATG7 *reduction on autophagy suppression were also confirmed by Western-blot^[^^21^^]^. MSCs were also treated with 500 ng/ml rapamycin (InvivoGen, USA), a well-known inducer of autophagy dissolved in dimethyl sulfoxide (Sigma, USA), in advance. The induction and suppression of autophagy in different MSC groups were confirmed by Western-blot analysis. Next, the autophagy-modulated MSCs were cultured under serum deprivation as well as hypoxic and oxidative stress conditions according to the standard protocols followed by WST assay^[^^21^^]^. 

***In vivo*** **studies**

***The production of an experimental model of acute liver failure***

ALF was induced in 8-week-old NMRI mice using **carbon tetrachloride **(CCl_4_, Merck, Germany), dissolved in olive oil. NMRI mice (25±2 g) were obtained from Animal Laboratory of Tehran University of Medical Sciences (Tehran, Iran) and kept in standard conditions in terms of light, food and water accessibilities. Animal experiments were conducted in accordance with the Institutional Animal Care and Use Committee of the Iran University of Medical Sciences. All protocols were conducted in accordance with the Ethics Committee of Iranian Blood Transfusion Organization (Tehran, Iran). 

Mice were divided into seven groups, each containing 10 mice. To establish the best ALF-induced method and to set up the optimized CCl_4_ dose, different concentrations of CCl_4 _(0.5, 1, 1.5, 2 and 2.5 ml/kg in olive oil) were intraperitoneally injected into different mice groups. Two control groups each containing 10 mice only received olive oil. After 24 h, blood and liver samples were collected. Induction of ALF was confirmed by biochemical evaluation of aspartate aminotransferase (AST) and alanine aminotransferase (ALT) of serum and histo/histopathological assessment of liver sections. The same number of mice (n=10) injected with different doses of CCl_4_ were included in order to determine the survival rate of mice during one week.

**Mesenchymal stem cells transplantation**

In order to investigate the MSCs regenerative effects, ALF-induced mice were divided into four groups, each containing 10 mice. ALF-induced mice in the first group were injected intravenously with autophagy-modulated MSCs (ALF-MSC-shRNA 3), in the second group, with normal MSCs (ALF-MSC) and in the third group with PBS (sham), receiving no cell therapy. Normal mice transplanted with normal MSCs (Normal-MSC) were considered as the fourth group.  1-1.3×10^6^ of different MSC groups were separately suspended in 200 µl PBS and intravenously administrated to each mouse via tail vein. All the MSCs transplantations were performed 24 h after ALF induction with 1.5 ml/kg CCl_4_ dissolved in olive oil. Immunosuppression was achieved by intraperitoneal injection of 10 mg/kg cyclosporine A to prevent any transplant rejection or immune reactions. Blood samples and liver tissues were collected at several time intervals (24, 48 and 72 h) after MSCs transplantation. Regenerative effects of different MSCs were evaluated through biochemical and histo/histopathological assays. The same numbers of transplanted mice (n=10) were included to determine the mice survival rates throughout one week after the administration of different MSCs groups.

**Biochemical assay**

AST and ALT, as the most important liver enzymes, were measured to determine liver functions. Twenty four hours after ALF induction and 24, 48 and 72 h after MSCs transplantation, blood samples were collected from the hearts of the mice under deep anesthesia with a cocktail of ketamine (150 mg/kg) and xylazine (15 mg/kg) (Alfasan, Netherlands). The ALT and AST serum levels were measured using an automatic analyzer (BT 3000 PLUS, Italy). 

**Histo/histopathological assay**

Liver tissue sections were analyzed in order to detect tissue injury and necrosis. The mice were sacrificed 24 h after ALF induction and 24, 48 and 72 h after MSCs transplantation. Liver tissues were collected and dissected in blocks. Then the samples were fixed with 10% formalin (Merck, Germany) and transferred to a tissue processor (Leica Biosystems, USA) for dehydration and further paraffin embedding. Liver tissue blocks were cut by a microtome (Leica Biosystems, USA) and stained with hematoxylin and eosin (H&E) (Sigma, USA) according to the standard protocol. Liver sections were observed under a light microscope to determine fat degeneration, hemorrhage, inflammation and alteration in lobule integrity. Semi-quantitative necrosis was scored to four levels based on the intensity of necrosis and diffusion of the inflammatory cells. Scoring was employed in order to facilitate the interpretation of pathologic results. The four scored levels were interpreted as: Zero, no necrosis and inflammation; One, mild hepatocyte necrosis with mild inflammatory reaction; Two, diffuse hepatocyte necrosis and intralobular necrotic bridges along with inflammatory reaction; Three, complete destruction of lobules, diffuse hepatic necrosis along with diffuse interlobular inflammatory reaction. 

**Survival rate assay**

The survival rates were surveyed in 10 mice within seven days after ALF induction and MSCs transplantation. All animals were evaluated regularly in terms of their survival rates, and the data was also recorded.

**Statistical analysis**

The quantitative data were presented as mean±standard deviation (SD) and analyzed using SPSS statistical software version 19 (SPSS Inc., Chicago, IL, USA). One-way ANOVA (or chi-square) and analysis of variances were used to determine statistically significant differences (*P*<0.05).

## RESULTS

**Fig. 1:**
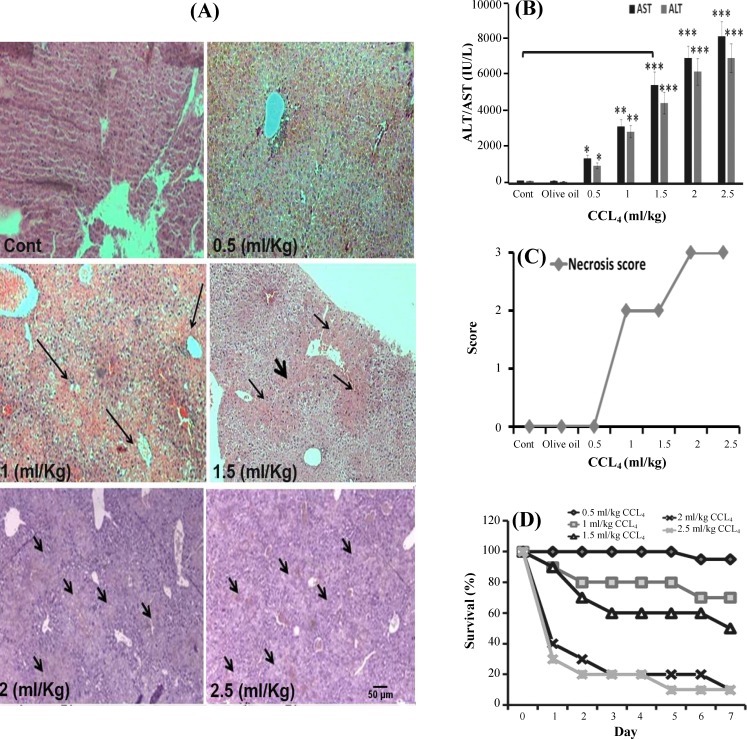
Confirmation of acute liver failure (ALF) induction in mouse models using different concentrations of CCl4. (A) Histo/histopatological assay. Liver samples were collected 24 h after treatment with 0.5, 1, 1.5, 2 and 2.5 ml/kg CCl_4_ in olive oil (three mice for each dose). Mice without any injection and mice injected with olive oil were considered as control. Multiple central lobular necroses (long narrow arrows) was induced using 1 ml/kg CCl_4_. The injection of 1.5 ml/kg CCl_4_ induced interalobular necrotic bridge (short thick arrow) and diffuse necrosis (medium narrow arrows). Severe diffuse necrosis and complete destruction of lobules (short arrows) were seen after the injection of higher CCl_4_ doses (H&E stain, magnification 40×, scale bar: 500 µm). (B) Biochemical assay of ALF-induced mice (three mice for each dose). Aspartate aminotransferase (AST) and alanine aminotransferase (ALT) were evaluated 24 h after the injection of 0.5, 1, 1.5, 2 and 2.5 ml/kg CCl_4_ in olive oil. ALT and AST serum levels were significantly higher in CCl_4_-received mice in comparison to the control groups (those without any injection and those that received olive oil alone). (mean±SD, *P≤0.05, **P≤0.01, ***P≤0.001). (C) The quantification of necrosis score in at least 30 microscopic fields. Administration of 1 and 1.5 ml/kg CCl_4_ induced type two necrosis but 2 and 2.5 ml/kg CCl_4_ led to type three necrosis. (D) The survival rate of ALF-induced mice. The survival rates of 10 ALF-induced mice were recorded during seven days after CCl_4_ injection. Also, >50% death was recorded in 2 and 2.5 ml/kg CCl_4_-treated groups. Altogether, 1.5 ml/kg CCl_4_ was chosen as the optimized ALF-inducing dose. Cont, control

CCl_4_ increased liver enzymes, changed liver histology and induced ALF. To provoke ALF, 0.5, 1, 1.5, 2 and 2.5 ml/kg CCl_4_ in olive oil were injected intrapritoneally into the mice. After 24 h, biochemical and histo/histopathological evaluations were performed to determine the optimized ALF-inducing CCl_4_ dose. Mice survival rate was also recorded during one week. As shown in Figure 1A, histopathological assay on liver sections indicated that the administration of CCl_4_ led to liver injury, and the integrity of liver tissue was changed due to hemorrhage and fat degeneration. Also, 1 ml/kg CCl_4_ generated mild inflammation in liver, while the injection of 1.5 ml/kg CCl_4_ induced intralobular necrotic bridges. The doses of 2 and 2.5 ml/kg CCl_4_ destructed lobules completely and caused diffuse necrosis with a hemorrhagic zone. There was no detected liver injury in the mice injected with olive oil as well as the mice without any injection (Fig. 1A). According to the biochemical tests, ALT and AST were increased significantly in the mice received 1.5 ml/kg CCl_4_ in comparison with non-injected mice (control group) (AST: 5910±503 *vs *138±32; ALT: 4980±460* vs *121±27) (*P*≤0.001). More hepatotoxicity was observed in massive ALT and AST release through more intensive liver injury for 2 and 2.5 ml/kg CCl_4 _(Fig. 1B). The quantification of necrosis score is presented in Figure 1C. Administration of 1 ml/kg CCl_4_ showed type one necrosis, while the injection of 1.5 ml/kg CCl_4_ induced type two necrosis in liver (as described above). The doses of 2 and 2.5 ml/kg CCl_4_ demonstrated type three liver necrosis, while there was no detected liver necrosis after the injection of 0.5 ml/kg CCl_4_ (Fig. 1C). The survival rate of ALF-induced mice was monitored for seven days after CCl_4_ injection. At lower CCl_4_ doses (e.g. 0.5 ml/kg), higher survival rate was recorded, while higher doses of CCl_4_ (2 and 2.5 ml/kg) led to more cases of death (90%) in treated groups (Fig. 1D). Altogether, after the interpretation of different test results, the dose of 1.5 ml/kg CCl_4_ was selected as the optimized dose of CCl_4_ to induce ALF. 

Suppression of autophagy in MSCs accelerates liver regeneration after ALF induction in shorter period of time. The isolated MSCs were characterized by flowcytometry and differentiation capacity analysis as described previously^[^^22^^]^. These cells expressed MSC markers and did not express hematopoietic markers. They were also able to differentiate into osteocytes, chondrocytes and adipocytes^[22]^. PCR and real-time PCR results confirmed the reduction of *ATG7* expression, and the suppression of autophagy was verified by Western-blot^[^^21^^]^. The results showed that the viability of autophagy-modulated MSCs was greater than those of control and autophagy-induced MSCs under severe stress conditions, such as oxidative stress, hypoxia and serum deprivation^[^^21^^]^. Then normal and autophagy-modulated MSCs were transplanted into ALF-induced mice. Also, two control groups were considered: 1) ALF-induced mice that received PBS (sham) and 2) normal mice that received normal MSCs (Normal-MSC).

**Fig. 2:**
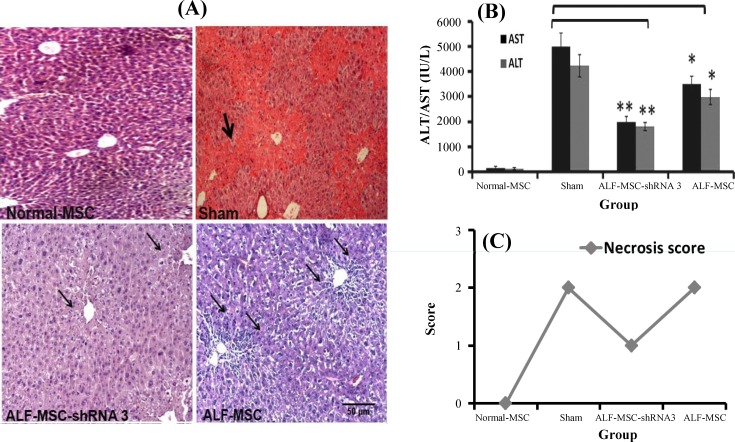
Therapeutic effects of different mesenchymal stem cells (MSCs) in ALF-induced mice 24 h after cell therapy. Normal MSCs and autophagy-modulated MSCs were transplanted in acute liver failure (ALF)-induced mice, and normal mice that received normal MSCs were considered as control. Their therapeutic potentialities were evaluated after 24 h. (A) Histo/histopathological assay. Accelerated repairing was observed in ALF-MSC-shRNA 3 mice received autophagy-modulated MSCs. Mild necrosis (narrow arrows) without interalobular necrotic bridge vs. persistent interalobular necrotic bridge (thick arrow) in mice that received no MSCs (sham group) and multiple diffuse necrosis in ALF-MSC that received MSCs without any autophagy modulation (H&E stain, magnification ×100, scale bar: 500 µm). (B) Biochemical assay. Aspartate aminotransferase (AST) and alanine aminotransferase (ALT) serum levels were reduced significantly in mice received autophagy-modulated MSCs (ALF-MSC-shRNA 3) and MSCs without any autophagy modulation (ALF-MSC) compared with sham. (mean±SD, *P≤0.05 and **P≤0.01). (C) Necrosis score quantification. Reduction of necrosis score from two to one was occurred after the administration of MSC-shRNA 3. There was no alteration of necrosis score in other groups

To evaluate the regenerative effects of the foregoing MSCs, biochemical and histo/histopathological analyses were performed after 24 h. Microscopic observation of H&E stained sections showed a mild necrosis and inflammation without any intralobular necrotic bridges that determined quick necrosis control or inhibition in ALF-MSC-shRNA 3 mice, which was received autophagy-modulated MSCs (Fig. 2A). However, more intensive necrosis was observed in sham and ALF-MSC, which was transplanted with normal MSCs without any autophagy modulation (Fig. 2A). As presented in Figure 2B, a sharp decline in liver enzymes was recorded in ALF-MSC-shRNA 3. ALT and AST serum levels were decreased significantly in ALF-MSC-shRNA 3 (AST: 1981±252; ALT: 1702±209) and ALF-MSC (AST: 3304±34; ALT: 2923±294) compared with sham (AST: 4970±481; ALT: 4093±409) (*P*≤0. 01 and *P*≤0. 05, respectively) (Fig. 2B). Necrosis score quantification also revealed that inflammation and necrosis decreased from type two to type one in ALF-MSC-shRNA 3 in comparison to sham and ALF-MSC (Fig. 2C). 

**Fig. 3:**
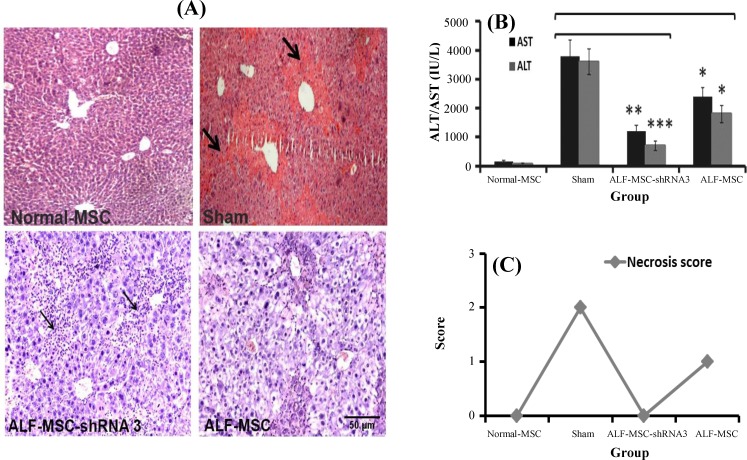
Therapeutic effects of different mesenchymal stem cells (MSCs) in acute liver failure (ALF)-induced mice 48 h after cell therapy. Normal MSCs and autophagy-modulated MSCs were transplanted in ALF-induced mice. Normal mice received normal MSCs and were considered as control. Their therapeutic potentialities were evaluated after 48 h. (A) Histo/histopathological assay. There was no significant necrosis in ALF-MSC-shRNA 3, and inflammatory cells (narrow arrows) contributed to a progressive regeneration rate. Persistent diffuse necrosis and interalobular necrotic bridge (thick arrow) were observed in sham group (H&E staining, magnification 100×, scale bar: 500 µm). (B) Biochemical assay. Aspartate aminotransferase (AST) and alanine aminotransferase (ALT) levels were decreased continually. Their serum levels were significantly lower in ALF-MSC-shRNA 3 and ALF-MSC compared with sham (mean±SD, *P≤0.05, **P≤0.01, ***P≤0.001). (C) Necrosis score quantification. As shown in the figure, necrosis score was reduced from one to zero in MSC-shRNA 3 and from two to one in ALF-MSC. However, the necrosis score in sham group was two

The second blood and liver samples were collected 48 h after MSC transplantation. Transplantation of MSC-shRNA 3 in ALF-induced mice led to the accelerated liver regeneration and convenient necrosis control (no necrosis) in liver tissue; however, a mild increase in inflammatory cells was also observed. This matter indicated the process of progressive repairing (Fig. 3A). In contrast, there was no sign of necrosis decrease in sham (Fig. 3A). As shown in Figure 3B, the reduction of AST and ALT (especially ALT) continued, and it was significant in ALF-MSC-shRNA 3 in comparison with sham (AST: 1238±213 *vs *3678±382; ALT: 780±148* vs *3450±349) (*P*≤0.01 and* P*≤0.001, respectively). Less reduction of ALT and AST was observed in ALF-MSC (AST: 2609±295, ALT: 1780± 241) (*P*≤0.05) (Fig. 3B). The quantification of necrosis score showed a decrease in necrosis score (from one to zero in ALF-MSC-shRNA 3 and from two to one in ALF-MSC) compared to sham with type two necrosis (Fig. 3C).

**Fig. 4:**
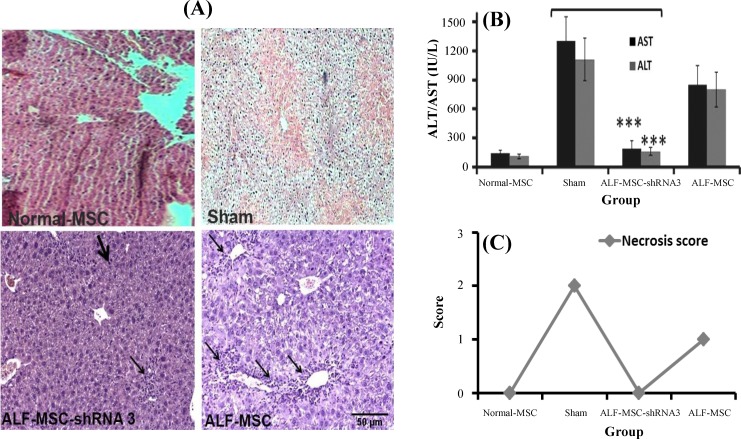
Therapeutic effects of different mesenchymal stem cells (MSCs) in acute liver failure (ALF)-induced mice 72 h after cell therapy. Normal MSCs and autophagy-modulated MSCs were transplanted in ALF-induced and normal mice that received normal MSCs were considered as control. Their therapeutic potentialities were evaluated after 72 h. (A) Histo/histopathological assay. A decreased number of inflammatory cells (narrow arrows) and the presence of repaired cells (thick arrow) were observed in ALF-MSC-shRNA 3. ALF-MSC (received MSCs without any manipulation) revealed increased inflammation and mild necrosis. Necrosis was observed in sham group (H&E stain, magnification 100×, scale bar: 500 µm). (B) Biochemical assay. Aspartate aminotransferase (AST) and alanine aminotransferase (ALT) levels approximately returned to the normal level in ALF-shRNA 3 compared with sham (mean±SD, ***P≤0.01). There was no significant difference between ALT and AST of ALF-MSC and sham. (C) Necrosis score quantification. Necrosis score was zero in MSC-shRNA 3 but the score was one in ALF-MSC and two in the sham group

**Fig. 5:**
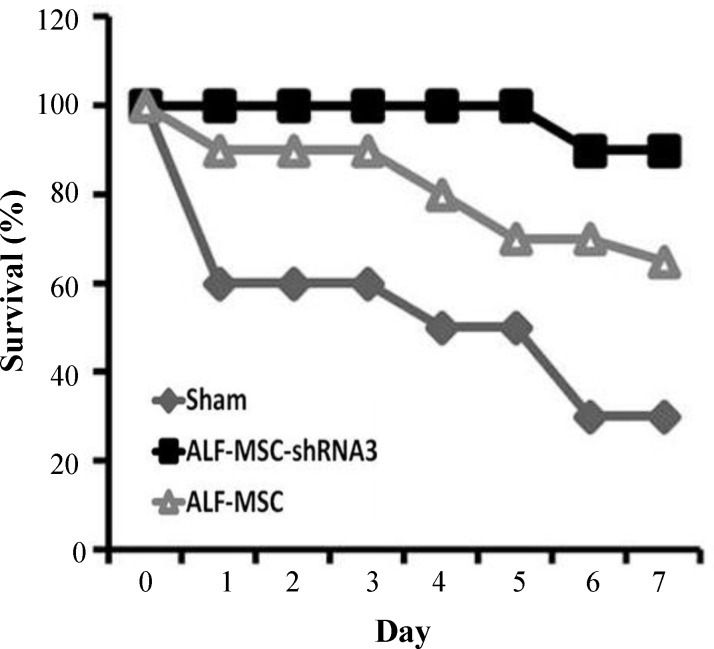
The survival rate of different transplanted mice. The survival rate of understudied mice was recorded during seven days of mesenchymal stem cells (MSCs) post transplantation. Approximately all ALF-induced mice transplanted with MSC-shRNA 3 (ALF-MSC-shRNA 3) stayed alive during this time period

Seventy two hours after MSCs transplantation, no necrosis and less inflammatory cells were observed in histo/histopathologicl liver sections of ALF-MSC-shRNA 3 mice; however, mild necrosis in ALF-MSC and multiple necrosis zones were detectable in sham (Fig. 4A). It is notable that the administration of autophagy-modulated MSCs not only inhibited necrosis progression in liver but also repaired the damaged tissue. Decreased AST and ALT levels were significantly different in ALF-MSC-shRNA 3 compared to those in sham (AST: 156±35 *vs *1316±200; ALT: 139±30* vs *1089±178) (*P*≤0.001). In other words, these liver enzymes returned approximately to their normal basal levels in ALF-MSC-shRNA 3. However, there was no significant difference between AST and ALT levels in ALF-MSC and sham (Fig. 4B). Notably, necrosis score zero (no necrosis) was also observed in ALF-MSC-shRNA 3, but in sham group, the necrosis score two was detected (Fig. 4C). The survival rates of mice were recorded during seven days after transplantation as presented in Figure 5. As shown in this Figure, 90% of ALF-MSC-shRNA 3 survived at the end of this time period compared to other groups. This result was in consistent with biochemical and histophatological findings and thus confirmed more regenerative potentials of MSC-shRNA 3 in ALF-induced mice. 

## DISCUSSION

More recently, our* in vitro *results showed that the suppression of autophagy in MSCs rendered these cells to be more robust under unfavorable micro-environments^[^^21^^]^. Based on MSCs promising characteristics, xeno-transplantation, allo-transplant-ation and auto-transplantation of MSCs with or without any modifications have been employed for the treatment of different diseases^[^^23^^]^. In allogeneic studies using mouse models, MSCs are derived from mouse, and then transplanted into mouse with less major histocompatibility complex mismatch. However, the major limitations include the differential characteristics, *in vitro* expansion and biological properties along with different immunosuppressive mechanisms of murine MSCs compared to human MSCs^[^^24^^]^. In this study, human MSCs were employed. Human MSCs derived from different tissues can readily be expanded *ex vivo*. Indeed, robust *in vitro* proliferation properties of human MSCs make them attractive therapeutic agents to be used in clinical trials^[^^24^^]^. Thus, preclinical animal studies with murine MSCs cannot be considered as an exact replica of human MSC-based clinical trials. However, human MSCs might be more promising to be employed in clinical trials. 

Herein, experimental ALF-induced mouse models were prepared using CCl_4_^[^^25^^]^, and then the therapeutic potentials of autophagy-modulated MSCs were investigated in these models. 

CCl_4 _is metabolized by P450 cythocrome. As a result, trichloromethyl free radicals (CCl_3_ and CCl_3_O_2_) and other reactive oxygen species are produced that finally induces ALF^[^^26^^]^. In the present study, transplantation of autophagy-modulated MSCs (MSC-shRNA 3) resulted in ALT and AST reduction, acceleration of liver regeneration and more increased survival rate in ALF-MSC-shRNA 3 mice compared with sham and ALF-MSC mice. In ALF and other acute diseases, it is very important to employ suitable medical interventions to save the time and patient’s life. Enhanced survival rate in MSC-shRNA 3 accelerated their regenerative potentialities in ALF mice during the first 3-4 days after transplantation. This result might be due to paracrine effects of MSC-shRNA 3, which is probably more efficient than their differentiation or other potentialities in term of liver regeneration^[^^27^^]^. MSCs produce and secrete a wide range of cytokines, chemokines and growth factors, including IL-6 (interleukine-6) and IL-10, which are involved in tissue regeneration^[^^28^^]^. IL-6 has protective effects on liver in experimental ALF models^[^^29^^,^^30^^]^ and triggers hepatocyte proliferation^[^^31^^]^. Anti-inflammatory and immunosuppressive functions of IL-10 might contribute to control necrosis and promote repairing process^[^^30^^]^. It seems that secreted growth factors from MSC-shRNA 3 stimulate liver de novo regenerative mechanisms and improve them to restore liver tissue in shorter period of time. In consistent with our findings, Cao *et al.*^[^^32^^]^ reported that the regenerative effects of placenta-derived MSCs in ALF-induced Chinese miniature pigs resulted in a higher survival rate due to the reduction of liver enzymes and necrosis. However, they used D-galactosamine to induce ALF in Chinese miniature pigs. In contrast to our findings, they reported that AST and ALT levels were reduced 96 h post MSCs transplantation. Shizhu *et al.*^[^^33^^]^ transplanted the bone marrow mononuclear cells in ALF-induced mice. This experiment resulted in a decrease in the level of liver enzymes more than one week after cellular injection. Bone marrow-derived mononuclear cells are heterogeneous cell population and might contain less potential cells for cell therapy purposes; moreover, it requires invasive sampling methods. 

In one study, MSC-derived hepatocytes were transplanted in acetaminophen-induced ALF models to decrease liver injury^[^^34^^]^. It requires the induction of partial differentiation in MSCs and increases process. Moreover, acetaminophen is not specific or popular hepatotoxin for ALF induction. Ma *et al.*^[^^35^^] ^have reported genetic manipulation and transduction of MSCs with lentiviral vector containing chemokine *CXC receptor 4* gene. The overexpression of this chemokine receptor can ameliorate the liver regeneration after ALF induction. This strategy increased the migration and homing ability of MSCs into damaged liver and decreased liver enzymes and tissue injury. However, genetic manipulation with viral vectors might be associated with some concerns in terms of safety and immune responses^[^^36^^]^. Another study has indicated that the transplantation of hypoxia-preconditioned MSCs accelerates liver regeneration due to increased vascular endothelial growth factor and serum albumin along with faster hepatocyte proliferation after hepatectomy^[^^37^^]^. 

In summary, in this study, we introduced a new strategy to enhance MSC-based cell therapy in liver diseases, particularly ALF. Autophagy-modulated MSCs ameliorated liver regeneration more likely via paracrine effects in a short period of time. Further studies, including evaluation of *ATG7* expression during MSCs expansion, its effects on MSCs survival and tracking of autophagy-modulated MSCs in animal models after transplantation are required.
